# Surface Modification of Hetero-phase Nanoparticles for Low-Cost Solution-Processable High-k Dielectric Polymer Nanocomposites

**DOI:** 10.1021/acsami.2c19559

**Published:** 2023-01-24

**Authors:** Suman Mandal, Yanbei Hou, Mingqing Wang, Thomas D. Anthopoulos, Kwang Leong Choy

**Affiliations:** † Institute for Materials Discovery, University College London, Roberts Building, Malet Place, LondonWC1E 7JE, U.K.; ‡ KAUST Solar Center (KSC), Physical Sciences and Engineering Division (PSE), King Abdullah University of Science and Technology (KAUST), Thuwal23955-6900, Kingdom of Saudi Arabia; § Division of Natural and Applied Sciences, Duke Kunshan University, Kunshan, Suzhou, Jiangsu 215316China

**Keywords:** hetero-phase filler (HPF), polymer nanocomposites, functionalized nanoparticles, high-k dielectric, energy-storage device

## Abstract

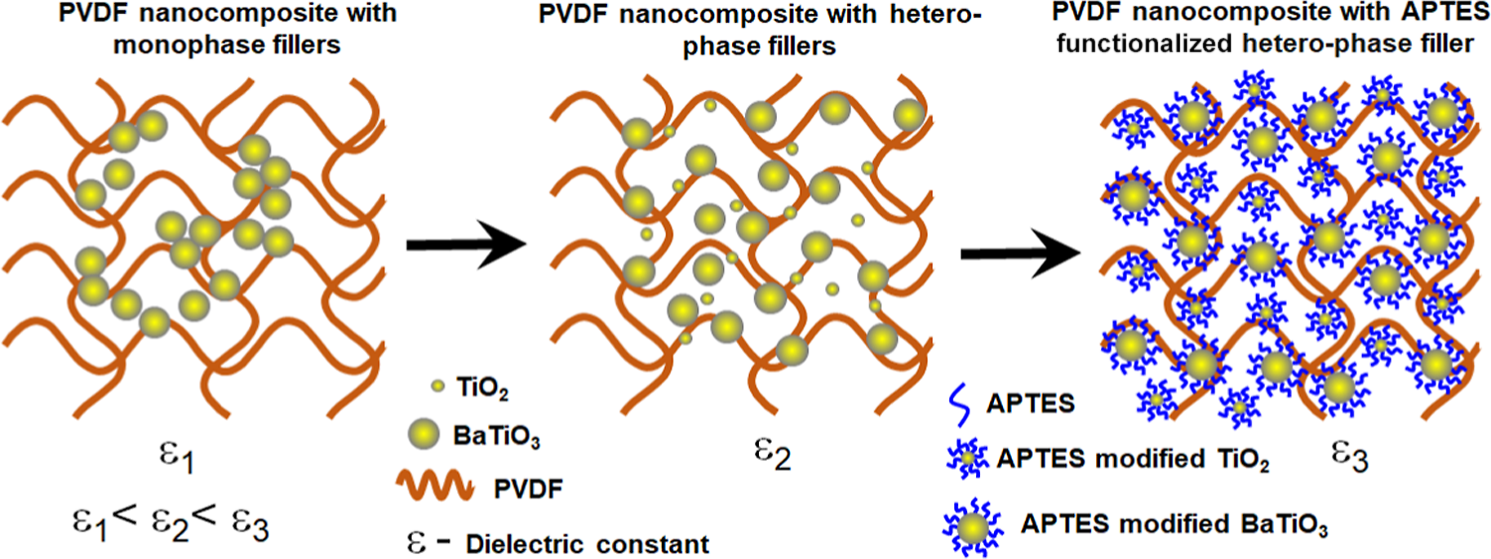

The surface modification of nanoparticles (NPs) is crucial for fabricating polymer nanocomposites (NCs) with high dielectric permittivity. Here, we systematically studied the effect of surface functionalization of TiO_2_ and BaTiO_3_ NPs to enhance the dielectric permittivity of polyvinylidene fluoride (PVDF) NCs by 23 and 74%, respectively, measured at a frequency of 1 kHz. To further increase the dielectric permittivity of PVDF/NPs-based NCs, we developed a new hetero-phase filler-based approach that is cost-effective and easy to implement. At a 1:3 mixing ratio of TiO_2_:BaTiO_3_ NPs, the dielectric constant of the ensuing NC is found to be 50.2, which is comparable with the functionalized BaTiO_3_-based NC. The highest dielectric constant value of 76.1 measured at 1 kHz was achieved using the (3-aminopropyl)triethoxysilane (APTES)-modified hetero-phase-based PVDF composite at a volume concentration of 5%. This work is an important step toward inexpensive and easy-to-process high-k nanocomposite dielectrics.

## Introduction

Ferroelectric polymer nanocomposites (NCs) have been widely studied in the last few decades mainly due to their exciting features such as easily processable, lightweight, cheap, and flexible nature that opens up various applications in the field of energy-storage capacitors,^1,2^ healthcare sensors,^3,4^ energy harvesters,^5,6^ field-effect transistors,^7^ and memory devices.^8,9^ Nowadays, the use of renewable energy sources such as solar, wind, and wave energy has increased significantly, and it successively increases the demand for environment-friendly, low-cost energy-storage devices that play an essential role in advanced electronics and electrical power systems. These are mainly batteries, electrochemical capacitors, and dielectric capacitors.^10−12^ Among them, the dielectric capacitor is used widely due to its unique features like ultrahigh power density, fast release of energy, and long lifetime. Materials with a high dielectric constant (ε_r_), a high break-down voltage, and low loss factors are essential for ideal dielectric-based energy-storage devices.^1,10,13^ However, dielectric capacitors suffer from low energy density, especially for applications requiring large capacitance with a small packaging size. The energy density (*W*) depends on the permittivity of the materials and the breakdown voltage (*V*_bd_), which is generally described by the following equation: , where  is known as the breakdown field strength of the device.^14^ In such systems, the aim is to enhance the dielectric’s permittivity while maintaining a high breakdown voltage. Conventional ceramic materials have shown higher dielectric constant values, excellent thermal stability, and environmental stability.^15−17^ However, the smaller breakdown strength, poor mechanical flexibility, and processing challenges have impeded their practical application in energy-storage devices.

Several polymers have been explored as dielectric materials in energy-storage capacitors due to their environment-friendliness, flexibility, and low-cost nature.^13,18,19^ However, the low dielectric constant of most polymers is often the main limiting factor even though the *V*_bd_ can be far superior to that of inorganic materials. For example, a dielectric capacitor based on biaxially oriented polypropylene (BOPP) shows a dielectric constant of circa 2 and a breakdown strength of 850 kV/mm.^1,20,21^ The ferroelectric polymer poly(vinylidene fluoride) (PVDF) is one of the superior candidates due to its higher dielectric constant (∼10) and dielectric strength of ∼800 kV/mm.^22^

In order to achieve the desired dielectric property with a higher dielectric constant, low tangent loss, and higher breakdown strength, polymer composites consisting of inorganic nanoparticles, conducting filler materials, and 2D materials embedded in a polymer matrix have attracted considerable attention.^14,23,24^ The polymer composites containing conducting filler materials show a higher dielectric constant, but the high tangent loss, low breakdown voltage, and difficulty in controlling the dispersion of the various components limit their application. Different oxide materials [TiO_2_, BaTiO_3_, SrTiO_3_, PbTiO_3_, and Pb(Zr, Ti)O_3_] with dimensions varying from microns to the nanoscale have been studied as fillers in the polymer matrix (e.g., PVDF, PVA, and PMMA).^14,18,25,26^ The ensuing composite systems show a relatively higher dielectric constant, moderate breakdown strength, and tangent loss, which make them attractive for application in energy-storage devices.^14,19,26^ The low filler loading, nano size, and relatively larger internal surface areas are the main advantages of these NC dielectrics. The formation of composites using a low filler loading in the polymer matrix helps to conserve almost all inherent and advantageous properties of the polymer, such as density, flexibility, and processability. Moreover, the large interfacial areas in the composite between the nanofiller phase and the polymer matrix promote exchange-coupling effects through the formation of a dipolar interface layer that improves the dielectric properties of the resulting NCs.^27^

Overall, polymer NCs combine the benefits of polymers with the functional properties of NPs, making them attractive for various applications. However, in the case of NP fillers, a high loading (>40%) is often required to achieve a relatively higher permittivity value.^14^ The incompatibility between organic and inorganic phases makes the preparation of homogeneous polymer composites challenging. In addition, the high surface energy of the NPs leads to inhomogeneous distribution and the formation of voids and pores inside the composite, which ultimately lowers the dielectric constant and increases the tangent loss of the composite dielectric. Another significant issue arises from the limited miscibility between the hydrophobic polymer matrix and the hydrophilic inorganic filler that leads to the non-uniform distribution of NPs within the polymer matrix. To address this, functionalized NPs have been introduced to enhance the polymer composite’s dielectric property by improving the homogeneity of the composite. Marder et al. have shown that phosphonic acid-modified BaTiO_3_-based polymethyl methacrylate (PMMA) NC can improve the dielectric constant of a polymer from 4.5 to a value of 11.^27^ Hydrogen peroxide and tetraflurophthalic acid-modified BaTiO_3_-based PVDF composites provided dielectric permittivities of 30 and 40, respectively.^28,29^ Yu et al. used NXT-105 for the surface modification of BaTiO_3_ and enhanced the dielectric permittivity of PVDF NC from 10 to 53.4.^30^ Functionalization of BaTiO_3_ using γ-aminopropyltriethoxy silane (KH550) and 3-glycidyloxypropyl) trimethoxysilane was also reported for the increment in the dielectric constant of PVDF composites.^31,32^ Dopamine modification of BaTiO_3_ nanoparticles was also reported to improve the dielectric constant of the PVDF-BaTiO_3_ composite.^33,34^ Polyvinylpyrrolidone (PVP)-modified BaTiO_3_ nanoparticles can also improve the dielectric permittivity of the NC film up to a value of 47.^35^ The reported dielectric constant and tangent loss for the raw BaTiO_3_ nanoparticles (diameter 100 nm) are circa 100 and 20, respectively.^36^ We have summarized the dielectric properties of various polymer composites in Table S1, where surface-modified NPs have been used as filler materials.

In this work, we functionalized TiO_2_ (⌀ ∼ 21 nm) and BaTiO_3_ (⌀ ∼ 100 nm) NPs with (3-aminopropyl) triethoxysilane (APTES) and blended them with PVDF to realize NC dielectric with improved characteristics. We successfully introduced the hetero-phase and surface-modified hetero-phase filler material-based polymer composites to further enhance their dielectric constant by using optimized mixtures of the functionalized TiO_2_ and BaTiO_3_ NPs. Nanocomposites with 3:1 mixing ratio of APTES-modified BaTiO_3_ and TiO_2_ within the PVDF matrix was found to yield the highest dielectric constant of 76.1. The approach is simple and creates new opportunities to develop inexpensive and simple to process high permittivity materials for a broad range of applications including gate dielectric of high-performance transistors.^37−39^

## Results and Discussion

**Figure 1:**
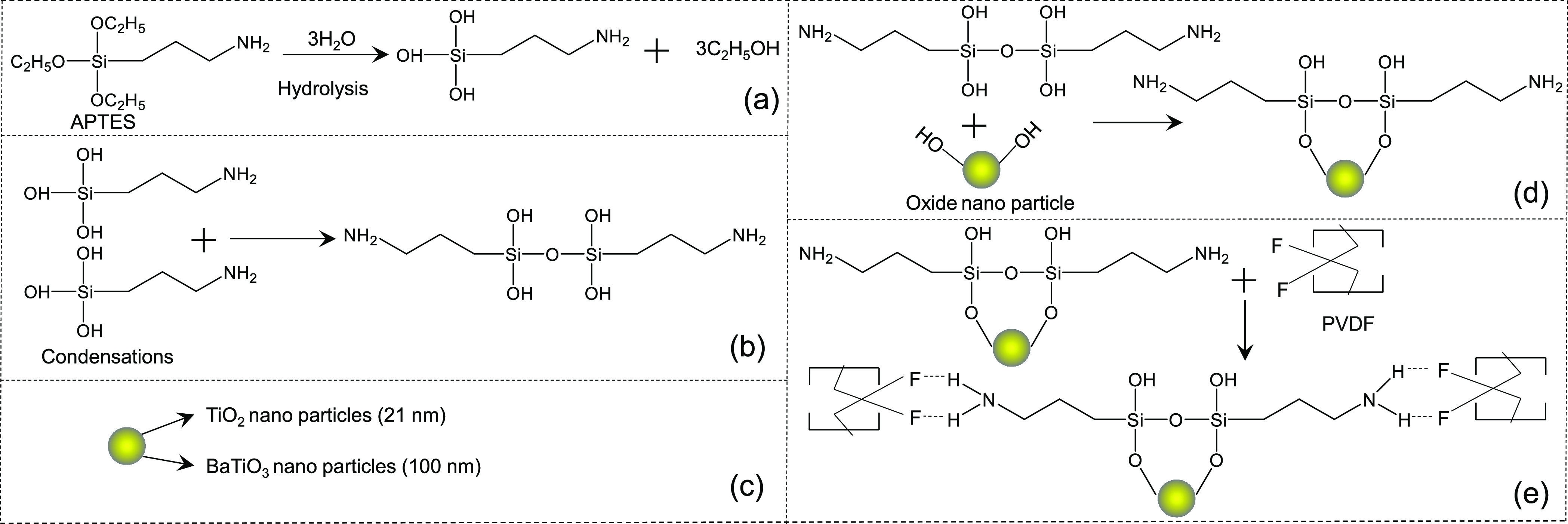
Schematic illustration of different steps for the formation of the functionalized nanocomposite. (a) Hydrolysis of (3-aminopropyl) triethoxysilane (APTES). (b) Condensation of the hydrolyzed product of APTES. (c) TiO_2_ (21 nm) and BaTiO_3_ (100 nm) nanomaterials before APTES modification. (d) APTES functionalization of TiO_2_ and BaTiO_3_ nanoparticles. (e) APTES-modified nanoparticles and PVDF composite formation.

In order to improve the dielectric property of the polymer NCs, we have functionalized the oxide NPs with APTES. The functionalization process is presented schematically in Figure 1. First, hydrolysis of APTES occurs followed by byproduct condensation,^40,41^ as shown in Figure 1a,b, respectively. Figure 1c shows a schematic of the TiO_2_ and BaTiO_3_ NPs with different diameters (21 and 100 nm, respectively), while the coupling of oxide nanoparticles with saline is presented in Figure 1d. Finally, oxide NPs functionalized with APTES are dispersed in the PVDF matrix uniformly aided by the interaction between the hydrogen atom on the surface of the NPs and the highly electronegative fluorine atom in the polymer (Figure 1e). The surface modification process steps are described in detail in the Experimental Section.

**Figure 2:**
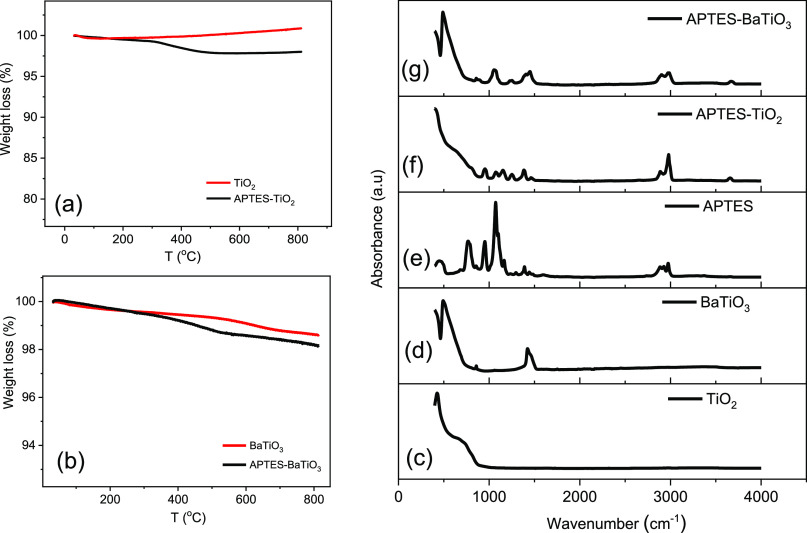
Percentage of weight loss during heating from 30 to 800 °C of (a) TiO_2_ and APTES-modified TiO_2_ (APTES-TiO_2_) and (b) BaTiO_3_ and APTES-modified BaTiO_3_ (APTES-BaTiO_3_). (c–g) FTIR spectra of TiO_2_, BaTiO_3_, APTES, APTES-modified TiO_2_, and APTES-modified BaTiO_3_ nanoparticles.

The thermal properties of the surface-modified oxide NPs were characterized using thermogravimetric analysis (TGA). For APTES-functionalized oxide NPs, a weight loss above 300 °C is observed and attributed to the decomposition of the organic silane-based coupling agents present on the NP surface. No such feature is observed for pristine oxide NPs. However, an upward tendency for pristine TiO_2_ was observed above 400 °C, and it may be due to the absorption of the gas molecules at high temperatures.^42^ However, after the degradation of APTES at high temperature, the residue present on the TiO_2_ nanoparticles may resist the absorption of gas molecules. As a consequence, the APTES-modified TiO_2_ nanoparticles do not show any upward tendency above 400 °C. The degradation process is more pronounced in APTES-modified TiO_2_ NPs (Figure 2a) compared with APTES-functionalized BaTiO_3_ NPs (Figure 2b). In order to further confirm the functionalization process, we have characterized the pristine and APTES-functionalized TiO_2_ and BaTiO_3_ NPs using FTIR spectroscopy. The corresponding spectra for TiO_2_, BaTiO_3_, APTES, APTES-modified TiO_2_ (APTES-TiO_2_), and APTES-modified BaTiO_3_ (APTES-BaTiO_3_) are given in Figure 2c–g, respectively. In Figure 2c, the peaks below 700 cm^–1^ are assigned to the Ti–O and Ti–O–Ti bonding of TiO_2_.^43^ In BaTiO_3_, the strong absorption peak around 570 cm^–1^ relates to the Ti–O stretching mode^44^ seen in Figure 2d. The bands present at 3400 cm^–1^ in Figure 2c,d are attributed to the stretching mode of surface hydroxyl (O–H) groups and possibly absorbed water molecules on the surface of NPs. In Figure 2e, the peaks at 3370 and 3295 cm^–1^ indicate the asymmetrical and symmetrical vibrations of N–H. Moreover, the peaks at 2928 and 2870 cm^–1^ arise from the asymmetrical and symmetrical stretching vibrations of the C–H bond in CH_2_ (Figure 2e). Asymmetrical stretching vibration of the C–H bond in CH_3_ is also observed at 2975 cm^–1^.^45^ The absorption at 1100 cm^–1^ occurs from the asymmetrical vibration of Si–O–C. The hydroxyl peak arises after the modification of TiO_2_ and BaTiO_3_ nanoparticles at 3660 and 3670 cm^–1^, concluding the functionalization process. It has been shown in Figure 2f,g. FTIR spectroscopy of PVDF is presented in Figure S1.

The morphology of pristine TiO_2_ and surface-functionalized TiO_2_ NPs has been studied using SEM. TiO_2_ NPs show a quite similar surface morphology to APTES-modified ones (Figure S2a,d). In order to verify the functionalization process, energy-dispersive X-ray spectroscopy (EDX) was used to analyze the composition of NPs. Figure S2b,c shows the element mapping of titanium (Ti) and oxygen (O) of pristine TiO_2_ NPs. As expected, the presence of carbon (C), silicon (Si), and nitrogen (N) together with titanate and oxygen are detected in APTES-functionalized TiO_2_ NPs (Figure S2e–i).

Similar to TiO_2_ NPs, BaTiO_3_ NPs are also known to agglomerate. To prevent this, we functionalized BaTiO_3_ NPs with APTES before blending them with PVDF. The morphology of the purchased BaTiO_3_ NPs is shown in Figure S3a. Evidently, the BaTiO_3_ NPs appear agglomerated. To improve the quality of the dispersion, we sonicated it vigorously for 30 min at room temperature. Figure S3b–d presents the mapping of barium, titanate, and oxygen elements in the NPs, respectively. The morphology of the APTES-functionalized BaTiO_3_ NPs is shown in Figure S3e, while the distribution of barium, titanate, oxygen, carbon, silicon, and nitrogen in those NPs is shown in Figure S3f–k.

**Figure 3:**
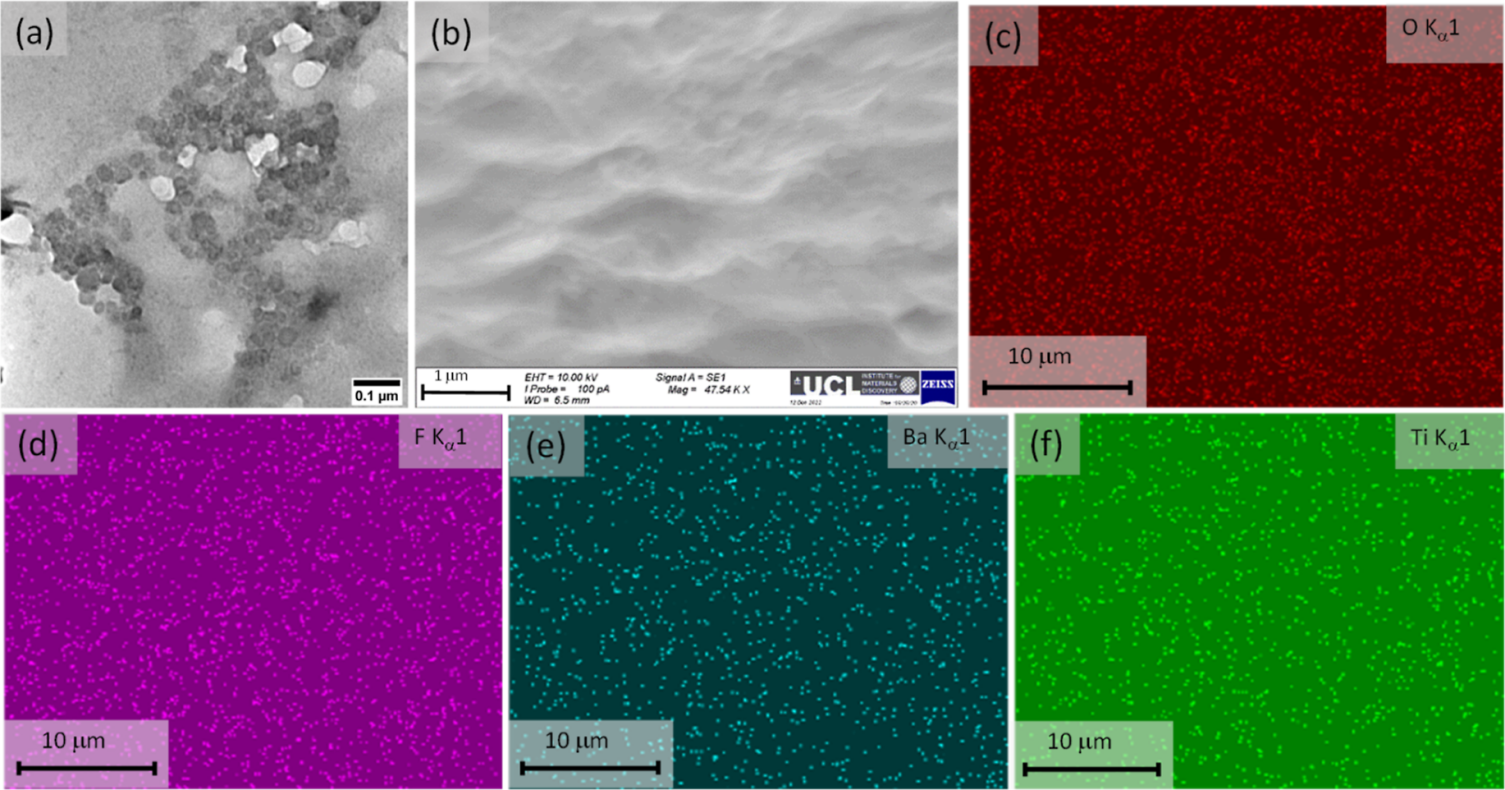
Surface morphology filler and composite materials. (a) TEM image of APTES-modified hetero-phase filler with 3:1 combination of TiO_2_ and BaTiO_3_ nanoparticles. (b) SEM image of the cross-section of APTES-modified hetero-phase filler (5% vol)-based PVDF nanocomposite. EDS elemental mapping of the film surface showing (c) carbon, (d) fluorine, (e) barium, and (f) titanium.

The surface morphology of APTES-modified hetero-phased filler was studied using transmission electron microscopy (TEM), and it is shown in Figure 3a. The TEM image of the hetero-phase filler shows functionalized nanoparticles with different dimensions. TiO_2_ nanoparticles with a dimension of around 20 nm are showing lower contrast in comparison to the BaTiO_3_ (<100 nm) nanoparticles. The cross-sectional SEM image of APTES-modified HPF-based PVDF composite of 5% vol concentration is shown in Figure 3b. It has been found that the surface morphology is uniform throughout the film. In order to confirm the presence of different elements in the HPF-based PVDF composite (5% vol), we have performed the surface elements mapping of the composite using EDS. The mappings of individual elements such as oxygen, fluorine, barium, and titanium are given in Figure 3c–f, respectively.

**Figure 4:**
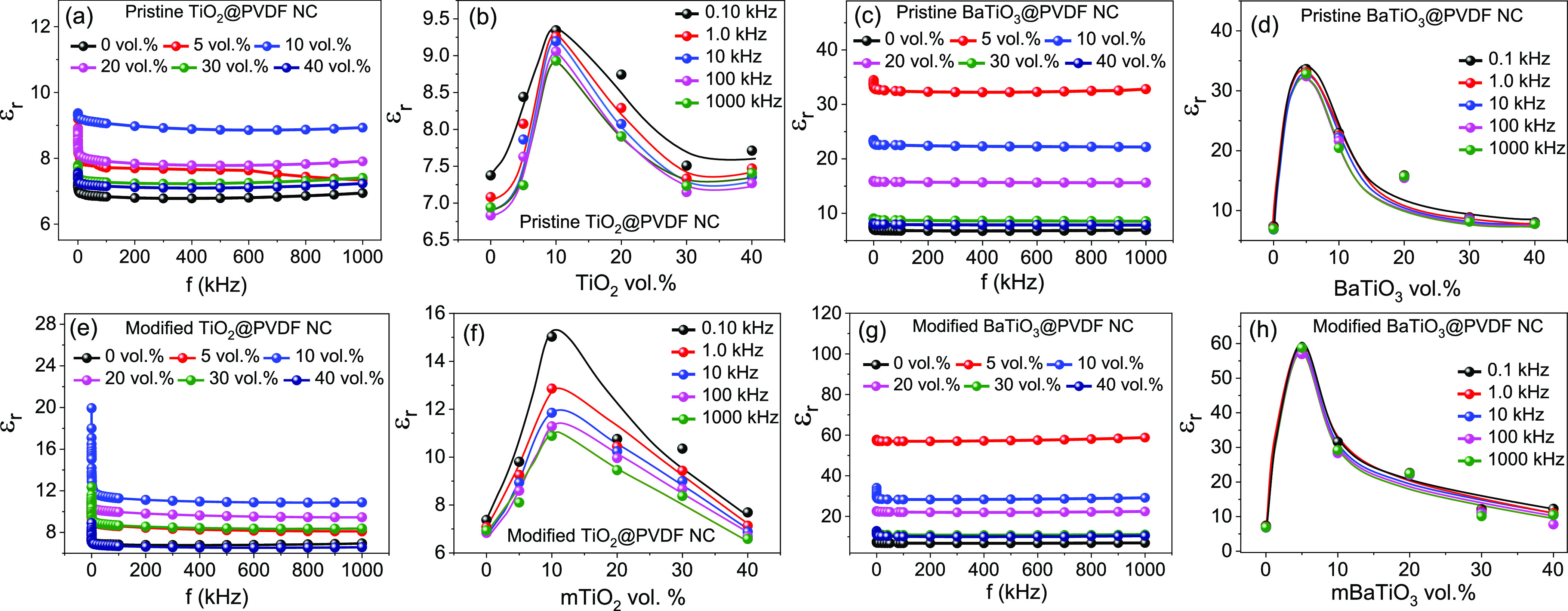
Frequency-dependent dielectric constant of PVDF nanocomposite with different oxide nanofillers: (a) TiO_2_ (21 nm), (c) BaTiO_3_ (100 nm), (e) APTES-modified TiO_2_, and (g) APTES-functionalized BaTiO_3_. Variation of the dielectric constant at different volume concentrations of the oxide nanofiller materials (b) TiO_2_ (20 nm), (d) BaTiO_3_ (100 nm), (f) APTES-modified TiO_2_, and (h) APTES-functionalized BaTiO_3_, indicating their percolation threshold.

Next, we fabricated a free-standing PVDF-based composite film utilizing pristine and APTES-modified TiO_2_ and BaTiO_3_ NPs. The details of the fabrication process are described in the Experimental Section. A PVDF-TiO_2_ polymer NC was prepared by mixing pristine TiO_2_ NPs in different volume concentrations (vol %) varying from 5 to 40%. The frequency-dependent dielectric constant variation for different volume concentrations (i.e., 5, 10, 20, 30, and 40%) are shown in Figure 4a.

Increasing the concentration of TiO_2_ NPs above 10% vol leads to NP agglomeration within the PVDF. The dielectric constant of the NC initially increases with the increasing amount of TiO_2_ NPs and reaches a maximum value at 10% vol concentration followed by a characteristic reduction. The dielectric constant of NCs with 10% vol of NPs measured at 1 kHz is 9.2. The corresponding tangent loss for the NC increases due to the agglomeration effects of TiO_2_ NPs. The tangent loss at a different frequency for these polymer NC materials is shown in Figure S4a. The percolation threshold for this PVDF-TiO_2_ polymer NC is 10% vol. As can be seen in Figure 4b, the percolation threshold depends only on the concentration of the filler materials. However, when TiO_2_ is replaced with BaTiO_3_ (100 nm), the dielectric constant increases due to the higher dielectric permittivity of BaTiO_3_ NPs compared to that of TiO_2_ NPs. The dielectric constant evolution at different frequencies and vol % of BaTiO_3_ NPs is shown in Figure 4c. The highest dielectric constant achieved is 32.8, measured at 1 kHz for a 5% vol of BaTiO_3_ NPs. The percolation threshold of the BaTiO_3_ NPs is 5% vol (Figure 4d). The lower percolation threshold of BaTiO_3_ is attributed to a larger diameter compared to TiO_2_ NPs. The frequency-dependent dielectric loss for these NCs is presented in Figure S4b.

The frequency-dependent dielectric constant variations of the APTES-modified TiO_2_ and BaTiO_3_ NPs-based polymer composites for different vol % are presented in Figure 4e,g, while the variation of the tangent loss is shown in Figure S4c,d. The highest dielectric constants for APTES-modified TiO_2_ and BaTiO_3_-based composite materials are 11.3 and 57.2, respectively. However, the percolation threshold of the polymer composite remains unchanged before and after functionalization with APTES for both oxide NPs. This is evidenced in the vol %-dependent dielectric permittivity for the pristine and modified TiO_2_ and BaTiO_3_ NPs-based NCs, shown in Figure 4b,f,d,h.

**Figure 5:**
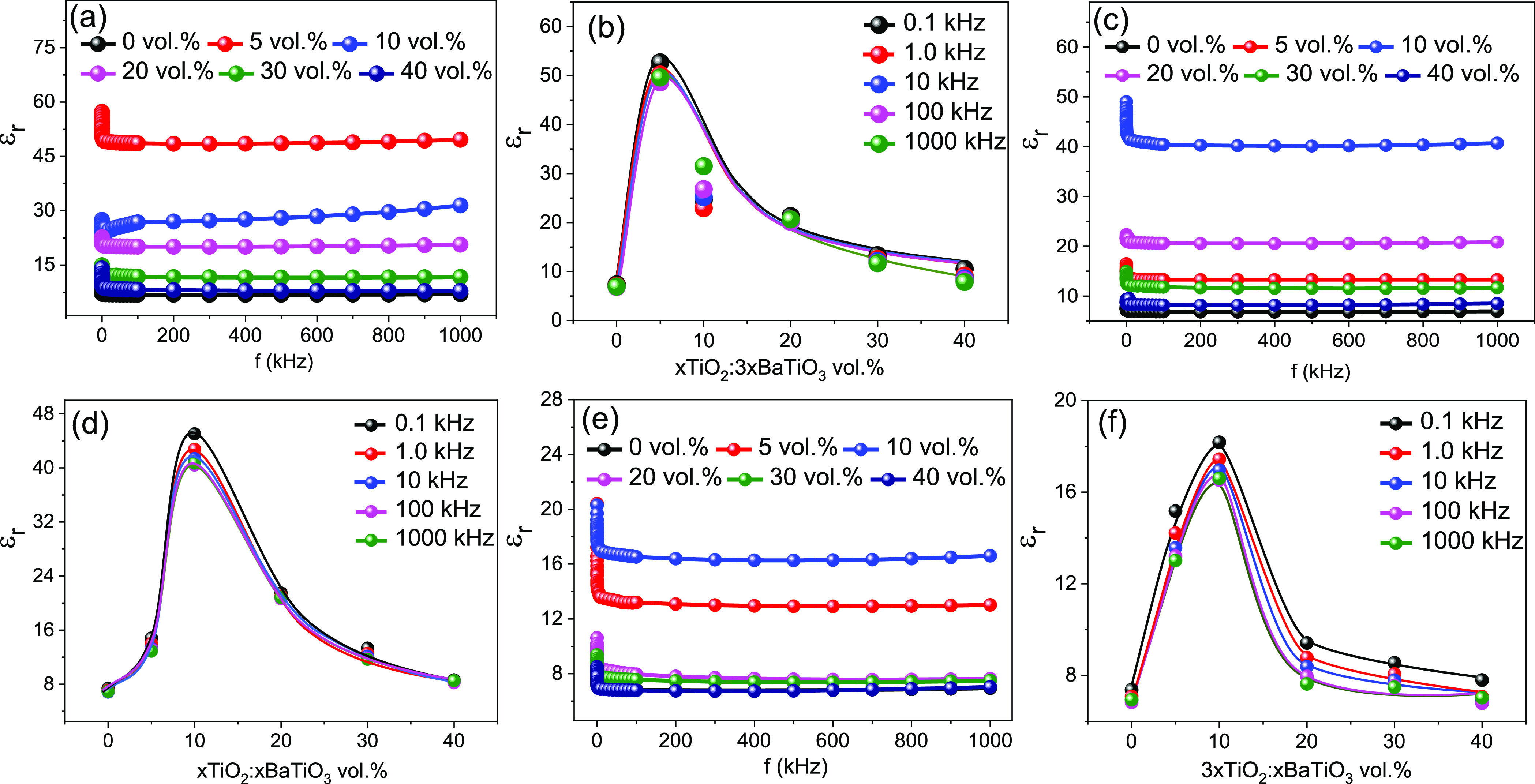
Dielectric constant variations with the frequency of hybrid oxide nanofiller-PVDF composite with TiO_2_ and BaTiO_3_ mixed in a ratio of (a) 1:3, (c) 1:1, and (e) 3:1. Change in dielectric constant values at different volume concentrations (i.e., 5 to 40%) of the hybrid oxide nanofiller mixed with a ratio of (b) 1:3, (d) 1:1, and (f) 3:1.

Next, we developed a hetero-phase filler (HPF) material using the oxide NPs and introduced it to PVDF in order to further improve the dielectric permittivity of the NCs. The filler materials TiO_2_ (21 nm) and BaTiO_3_ (100 nm) have been mixed in different weight ratios of 1:3, 1:1, and 3:1 to produce the HPF (TiO_2_/BaTiO_3_) before being dispersed in DMF. The volume concentration of the HPF component with respect to PVDF was varied from 5 to 40% (5, 10, 20, 30, and 40%) to produce the different NCs. The frequency dependence of dielectric constant variations of the various NCs of different mixing NP ratios of 1:3, 1:1, and 3:1 at different volume concentrations are shown in Figure 5a,c,e, respectively. Interestingly, we have observed that the NC made with the HPF with a mixing ratio of 1:3 shows the highest dielectric constant of 50.2 at 1 kHz, which is comparable to the functionalized BaTiO_3_ filler-based composite (ε_r_ = 57.2). This is due to the uniform mixing of HPF materials inside the PVDF matrix. It is also found that if we increase the amount of TiO_2_, the dielectric constant decreases. The highest dielectric constant measured at 1 kHz for the hetero-phase filler (TiO_2_ and BaTiO_3_) with a mixing ratio of 1:1 and 3:1 is 42.7 and 17.4, respectively. The volume concentration-dependent dielectric constant variation for these HPF materials with different mixing ratios of 1:3, 1:1, and 3:1 is shown in Figure 5b,d,f, respectively. It is visible from Figure 5b,d,f that the percolation threshold is dependent on the amount of TiO_2_ NPs as their mean diameter is much lower (∼5 times) than that of BaTiO_3_ NPs. The percolation threshold values of the HPF (TiO_2_/BaTiO_3_) with mixing ratios 1:3, 1:1, and 3:1 are 5, 10, and 10%, respectively. The tangent loss results for the combination of nanoparticles in the hetero-phase filler of TiO_2_ and BaTiO_3_ NPs with various ratios of 1:3, 1:1, and 3:1 based NCs are shown in Figure S5a–c, respectively.

**Figure 6:**
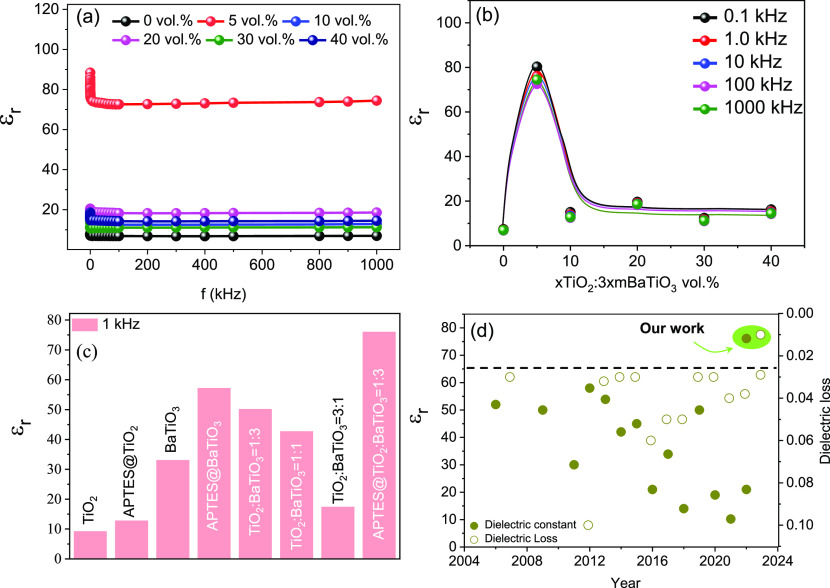
Frequency-dependent dielectric constant variation of the hybrid PVDF composite with APTES-functionalized TiO_2_ and BaTiO_3_ mixed with a ratio of 1:3. (b) Dielectric constant variation at different volume concentrations (5 to 40%) of the hybrid oxide nanofiller combined with a ratio of (a) 1:3. (c) Histogram plot comparing the dielectric constant values of PVDF nanocomposites with various filler materials at 1 kHz. (d) Comparison of state-of-the-art polymer nanocomposite with surface-modified nanoparticles as a filler material.

Finally, APTES-modified NPs were utilized to produce the HPF before mixing it into the PVDF matrix to produce the NC. The highest dielectric constant was observed in the polymer composite containing the HPF with TiO_2_/BaTiO_3_ in a 1:3 ratio for 5% vol. Figure 6a shows the frequency-dependent dielectric properties of the APTES-modified hetero-phase nanoparticle-based polymer composite for various vol of the filler materials. The highest measured dielectric constant for these polymer composites at a frequency of 1 kHz is found to be 76.1. The percolation threshold of the APTES-modified NCs remains the same as that of NCs based on HPF fillers of the pristine oxide NPs. Figure 6b shows the variation of the dielectric constant plotted with different volume concentrations of APTES-modified NPs. The dielectric loss for APTES-modified HPF-based PVDF composite is shown in Figure S5d. The variation of the experimentally measured dielectric constant values of the NCs with different fillers is displayed in a histogram plot in Figure 6c.

The surface topography images of the top and bottom surface of the free-standing APTES-modified TiO_2_ and BaTiO_3_ nanoparticles mixed in a ratio of 1:3 for various vol % in PVDF are shown in Figures S6a–f, S7a–f, respectively. The elements present in the energy-dispersive x-ray analysis (EDAX) spectra of the PVDF composite film further confirm the composition of the filler materials inside the NC. The EDAX spectra for PVDF films and the composite materials with HPF at vol of 5, 10, 20, 30, and 40% are shown in Figures S8–S13, respectively. The mechanical property test of the PVDF and APTES-modified hetero-phase filler of 3:1 of BaTO_3_/TiO_2_ NPs-based PVDF composite with 5% vol. concentration was carried out, and the corresponding stress–strain curves are shown in Figure S14a,b, respectively. The incorporation of the filler materials improved Young’s modulus a little bit from 9878.87 to 10720.19 MPa but decreased the extension from 2.22 to 1.89 mm. It implies that the addition of 5 vol % inorganic NPs into PVDF increased the strength a little but decreased the toughness of the polymer due to the agglomeration of NPs in the matrix.

The most significant developments in the dielectric properties of the high-k polymer NCs based on APTES-modified filler over the last 2 decades are summarized in Figure 6d. The maximum values of dielectric constant and minimum values of dielectric loss are considered for each year taken from the literature with the details given in the Supporting Information Table S1. The HPF-modified filler-based polymer nanocomposite shows significant improvement in the dielectric property of the polymer nanocomposite. Thus, we believe that the proposed hetero-phase filler concept enabled by simple surface functionalization of the NPs creates new opportunities for the development of printable high-k dielectrics. This material is suitable for a broad range of applications such as sensors, memory devices, thin-film transistors, and thin-film capacitors in an active matrix display due to their easy processing. Low-cost and lightweight make it suitable for applications in 5G communication as a substrate, base station, and coupler applications.

## Conclusions

We have systematically studied the dielectric properties of inorganic/organic nanocomposites based on TiO_2_ and BaTiO_3_ nanoparticles incorporated into a PVDF matrix. The percolation threshold of the TiO_2_ (10% vol)-PVDF nanocomposite yielded a dielectric constant of about 9.2 measured at a frequency of 1 kHz. The percolation threshold for the BaTiO_3_-PVDF nanocomposite decreased to 5% vol due to the larger diameter (100 nm) of the BaTiO_3_ NPs, yielding a maximum dielectric permittivity of 32.8. Modifying the surface of TiO_2_ and BaTiO_3_ NPs with APTES was found to improve their dispersibility within the PVDF. The resulting NCs exhibited optimized dielectric constant values of 11.3 and 57.2 for APTES-TiO_2_ and APTES-BaTiO_3_ NPs, respectively. By combining the APTES-TiO_2_ and APTES-BaTiO_3_ NPs, we developed a hetero-phase filler and studied its impact on the dielectric constant of PVDF-based NCs. The addition of 5% vol of APTES-TiO_2_ and APTES-BaTiO_3_ NPs with a mixed ratio of 1:3 led to NCs with a dielectric permittivity of 76.1 and a tangent loss of 0.01, measured at 1 kHz. The excellent performance of the HPF NCs is attributed to the synergy effect of the size and dielectric constants of the two nanofillers, which has led to the maximum interface and interphase polarization of the nanocomposite. The designed high-k NCs with low dielectric loss have promising applications in capacitors and gate dielectrics for large-area, thin-film electronics.

## Experimental Section

### Materials

Poly(vinylidene fluoride) (PVDF, 99.9%, MW = 690,000) powder was purchased from Alfa Aesar and used without further modification. N-dimethylformamide (DMF, 99%) was used as a solvent. Titanium dioxide (TiO_2_, >99.5%, diameter of 21 nm) and barium titanate (BaTiO3, >99%, diameter ≤ 100 nm) NPs purchased from Sigma-Aldrich were used as filler materials. (3-Aminopropyl)triethoxysilane (APTES, 99%) was used to functionalize the nanoparticles and was also obtained from Sigma-Aldrich.

### Functionalization of Nanoparticles

0.5 gm of each type of NPs (TiO_2_ and BaTiO_3_) was taken in two bottles containing 50 mL of DI water. The NPs were dispersed properly by sonicating them using a Fisher probe sonicator for 30 min. Subsequently, APTES was added dropwise in the dispersion. The solution was refluxed at 80 °C for 8 h during continuous stirring conditions. Then, the dispersed surface-modified particles were separated from the solvent by centrifuging the dispersion at 10,000 rpm for 10 min and redispersed in fresh DI water by sonicating it again before completing the washing with DI water in two different cycles. Finally, the functionalized particles were dried in an oven at 80 °C for 24 h.

### Free-Standing Composite Film Fabrication

PVDF was dissolved in DMF solvent by stirring it for 14 h using a magnetic stirrer at room temperature to prepare a concentration of 120 mg/mL. Then, the as-received and functionalized NPs (TiO_2_ and BaTiO_3_) were dispersed in DMF with a concentration of 5 mg/mL by sonicating the solution for 30 min. The dispersed NPs were added to the PVDF solution with different volume concentrations of 5, 10, 20, 30, and 40%. Subsequently, the mixture was stirred for 2 h at room temperature to disperse the particles in the polymer matrix.

Finally, the solution was placed in a Petri dish and dried at 80 °C to form the nanocomposite film peeled off from the Petri dish.

### Characterization of Nanoparticles and Free-Standing Film

The functionalized NPs were characterized by thermogravimetric analysis (PerkinElmer thermal analyzer, TGA 4000) and Fourier transform spectroscopy (PerkinElmer, Spectrum Two). The dielectric property of the free-standing NC film was characterized by IET 7600 plus an LCR meter with the composite film’s surface morphology, and chemical composition was studied using a scanning electron microscope (ZEISS) equipped with EDX. A Jeol 2100 transmission electron microscope was used to characterize the morphology of the functionalized mixed nanoparticles. Tensile test was carried out using OmniTest-5kN Universal Testing System from Mecmesin Ltd.

## Supporting Information Available

FTIR spectroscopic data and tangent loss, front and bottom side FESEM images of the free-standing PVDF-based NC film, and EDX spectra of the surface-modified hetero-phase-based NC film (PDF).
