# Supplementary material for: Surface Modification of Hetero-phase Nanoparticles for Low-Cost Solution-Processable High-k Dielectric Polymer Nanocomposites
Source: ACS Appl Mater Interfaces. 2023 Jan 24;15(5):7371–9. doi: 10.1021/acsami.2c19559 (PMC9923685; doi:10.1021/acsami.2c19559)
Supplement: Supplementary file 1 — am2c19559_si_001.pdf [file am2c19559_si_001.pdf]

Supporting Information

Surface Modification of Hetero-Phase Nanoparticles for Low-Cost Solution-Processable High-k Dielectric Polymer Nanocomposites

S. Mandal[1, 2]*, Y. Hou[1], M. Q. Wang[1]*, Thomas D. Anthopoulos[2], and K. -L. Choy[1,3]*

[1]Institute for Materials Discovery, University College London, Roberts Building, Malet, London WC1E 7JE, United Kingdom

[2] King Abdullah University of Science and Technology (KAUST), KAUST Solar Center (KSC), Physical Sciences and Engineering Division (PSE), Thuwal 23955-6900, Kingdom of Saudi Arabia

[3]Duke Kunshan University, Division of Natural and Applied Sciences, Jiangsu, China 215316

*E-mail: suman.mandal@kaust.edu.sa

kwang.choy@dukekunshan.edu.cn

mingqing.wang@ucl.ac.uk

The self-standing PVDF film has been characterized using FTIR spectroscopy, as shown in Figure S1. It has been observed from the FTIR spectroscopy that it mainly consists of three main phases of PVDF, which are α, β and γ phases. The characteristic peaks for α phase are present at 410 cm^{-1}, 488 cm^{-1}, 532 cm^{-1}, 614 cm^{-1}, 763 cm^{-1}, 795 cm^{-1}, 975 cm^{-1}, and 1423 cm^{-1}).[1] In the FTIR, the absorption bands at 507 cm^{-1}, 840 cm^{-1}, 875 cm^{-1}, 1071 cm^{-1}, 1279 cm^{-1}, and 1430 cm^{-1} are related to β phase of the PVDF polymer. The peaks observed at 431 cm^{-1}, 482 cm^{-1}, 840 cm^{-1}, 875 cm^{-1}, 1071 cm^{-1}, 1168 cm^{-1}, 1231 cm^{-1}, and 1420 cm^{-1} represent the characteristic bands of the γ phase. Therefore, the peaks at 840 cm^{-1}, 875 cm^{-1}, 1071 cm^{-1}, and 1430 cm^{-1}, can be related to both β and γ phases.

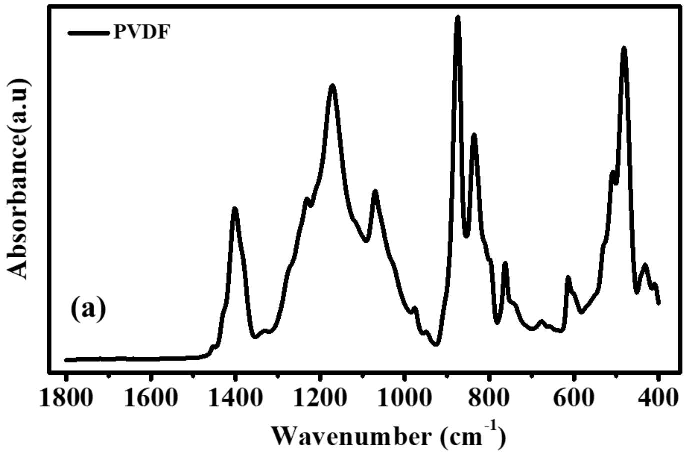

Figure S1. (a) FTIR spectroscopy of PVDF-based self-standing film.

SEM images of TiO_2 and APTES functionalized TiO_2 nanoparticles are presented in Figures S2a and S2d. The EDX mapping of different elements present in TiO_2 nanoparticles shows in Figures S2b and S2c. Figure S2 (e-i) represent elemental EDX mapping of surface-modified

TiO_2 nanoparticles, and it also confirms the APTES modification on the surface of TiO_2 nanoparticles.

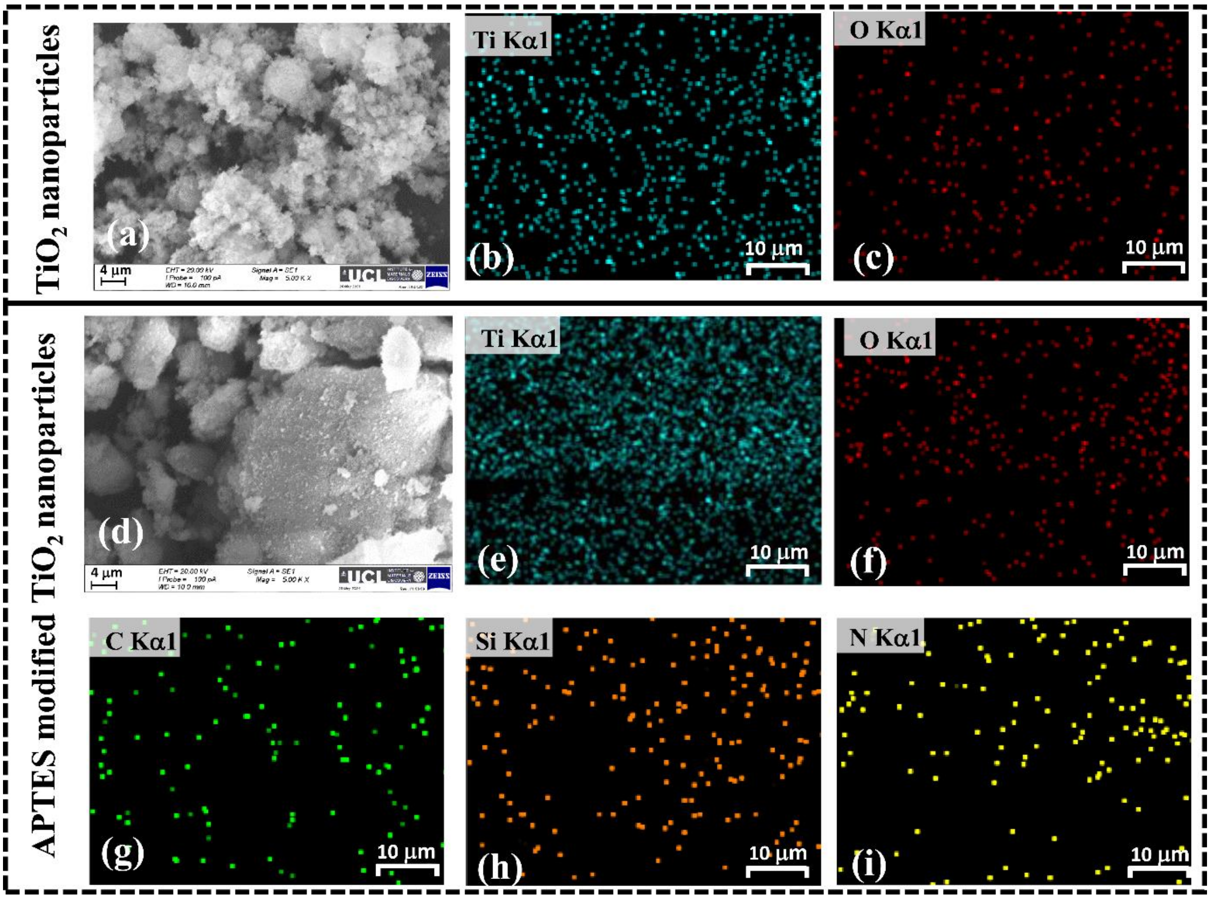

Figure S2. (a) presents the morphology of TiO_2 nanoparticles. (b), (c) depict EDX mapping of titanium (Ti) and oxygen (O) from TiO_2 nanoparticles. (d) represents the morphology of APTES-modified TiO_2 nanoparticles. (e), (f), (g), (h), and (i) illustrate the mapping of Titanium (Ti), Oxygen (O), Carbon (C), Silicon (Si), and Nitrogen (N) that present in the functionalized TiO_2 nanoparticles, respectively.

The surface morphology of $BaTiO_3$ and APTES-modified $BaTiO_3$ is presented in Figures S3a and S3e, respectively. The elemental EDX mapping of Ba, Ti and O atoms are shown in Figures

S3 (b-d), respectively. Similarly, EDX mapping of different elements present in the surface-modified $BaTiO_3$ is shown in Figure S3(f-k). It also confirms the surface functionalization of $BaTiO_3$ nanoparticles.

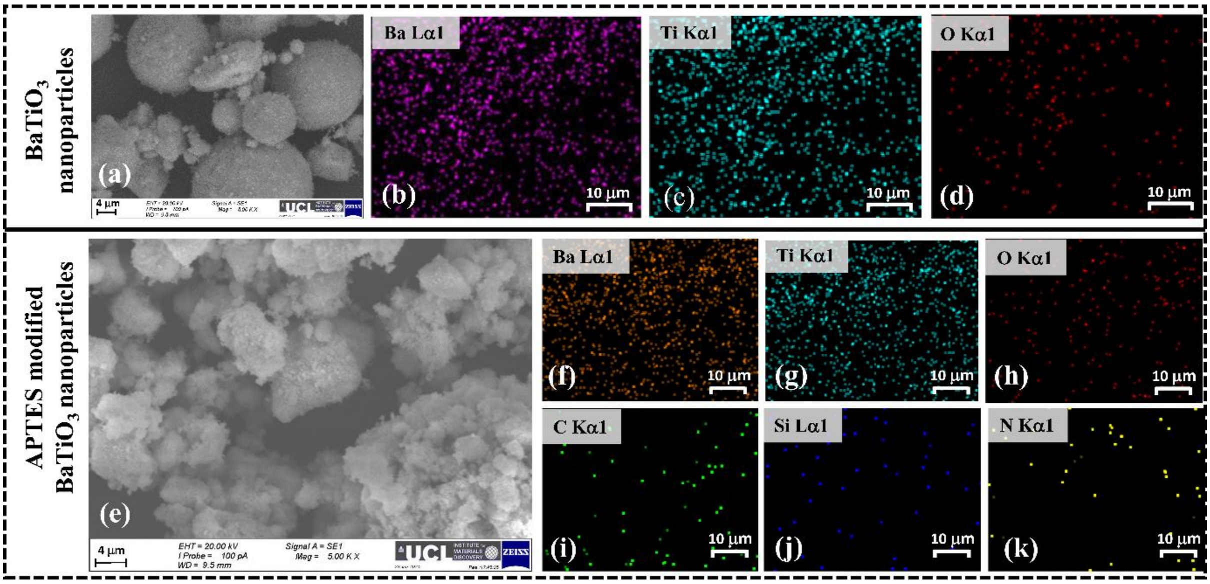

Figure S3. (a) Surface morphology of $BaTiO_3$ nanoparticles. (b), (c), (d) depict EDX mapping of barium (Ba) titanium (Ti) and oxygen (O) from $BaTiO_3$ nanoparticles. (e) represents the morphology of APTES-modified $BaTiO_3$ nanoparticles. (f)-(k) illustrates the mapping of barium, titanium (Ti), Oxygen (O), Carbon (C), Silicon (Si), and Nitrogen (N) that are present in the functionalized $BaTiO_3$ nanoparticles, respectively.

The tangent loss also has been characterized by the self-standing PVDF composite film. Figure S4a and S4b present the frequency-dependent dielectric loss considering the filler materials as TiO_2 and $BaTiO_3$, respectively. The tangent loss of APTES-modified TiO_2 and $BaTiO_3$ nanoparticles (NPs) based composite has been shown in Figures S4c and S4d, respectively. It has been observed that dielectric loss for surface-modified $BaTiO_3$ NPs-based composite has

lower dielectric loss among the other polymer composites. The measured dielectric loss at 1 kHz for APTES modified $BaTiO_3$-based composite is 0.037.

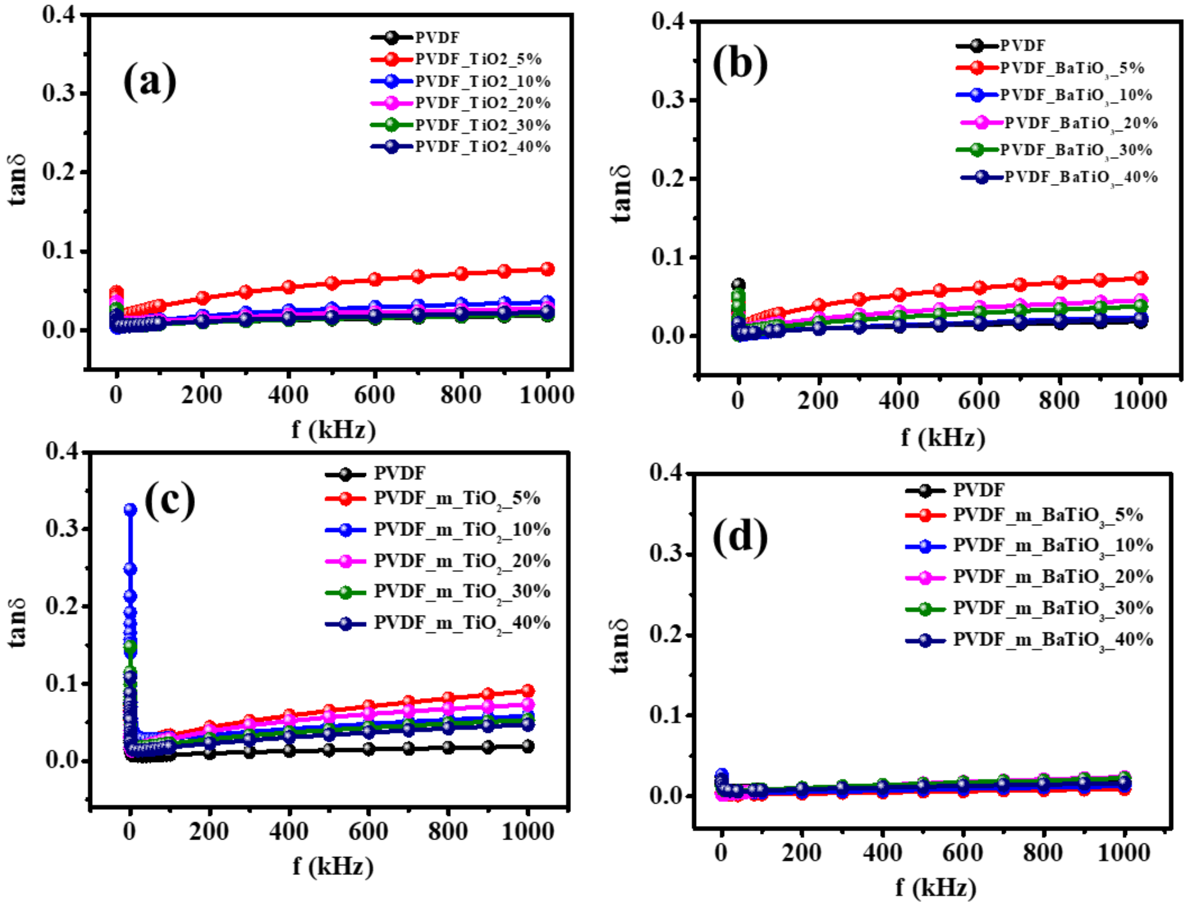

Figure S4. Tangent loss of the PVDF composite based self-standing film with different filler materials (a) TiO_2 (b) $BaTiO_3$ (c) APTES functionalized TiO_2 and (d) APTES functionalized $BaTiO_3$ at different volume concentrations varies from 5% to 40%, respectively.

The tangent loss of hetero-phase NPs based dielectric composite also has been studied. The dielectric loss for the combination of NPs in the hetero-phase filler of TiO_2 and $BaTiO_3$ with ratios of 1:3, 1:1 and 3:1 are shown in Figure S5 (a-c), respectively. The loss tangent for APTES-modified hetero-phase based polymer composite is shown in Figure S5d. It has been found that the dielectric loss is in the same order for all of this composite film. The measured

dielectric loss for APTES-modified hetero-phase filler with mixing ratio of TiO_2 and $BaTiO_3$ nanoparticles 1:3 based polymer composite is found 0.01. It is 3.7 times less than in comparison to $BaTiO_3$ based composite film.

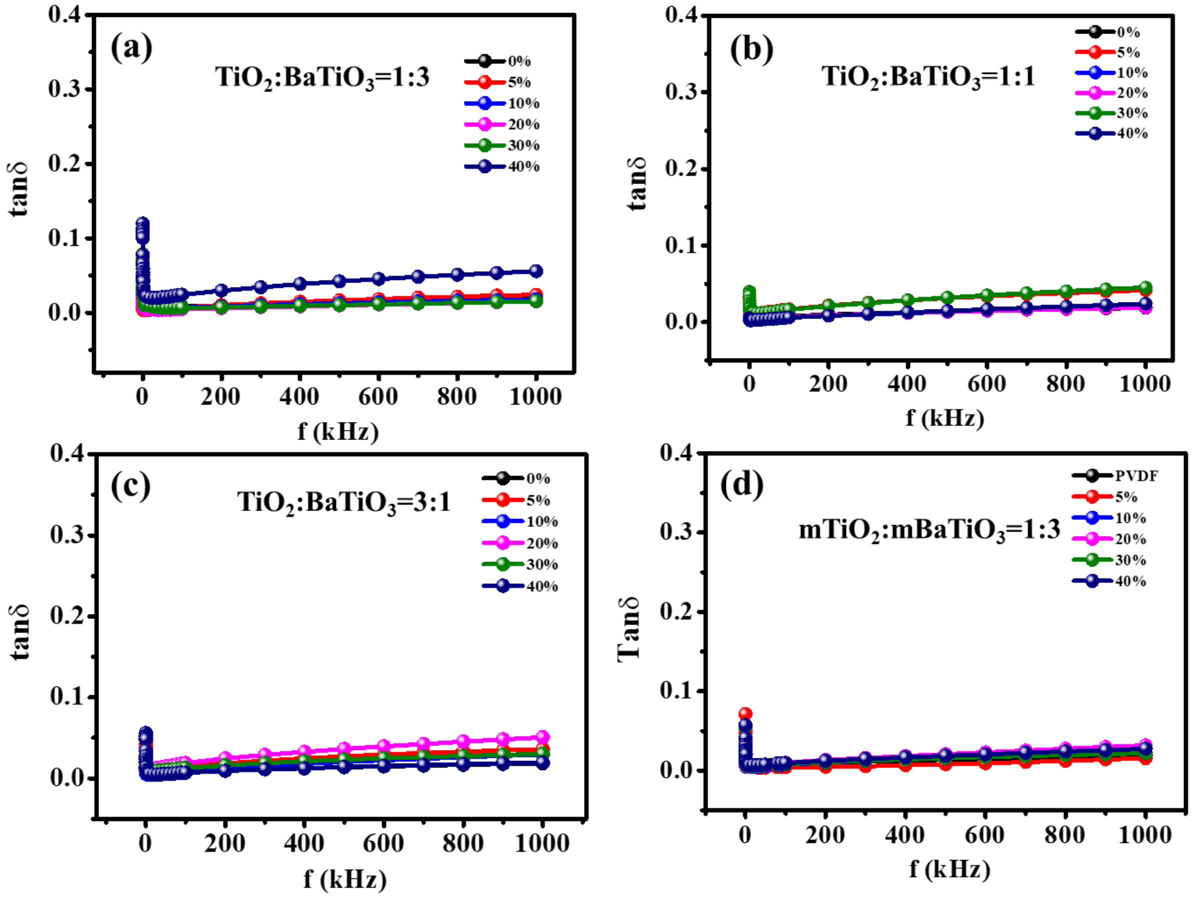

Figure S5. Tangent loss PVDF composite self-standing film of hetero-phase filler based on a combination of TiO_2 and $BaTiO_3$ nanoparticles (a) 1:3, (b) 1:1, and (c) 3:1, respectively. (d) APTES functionalized hetero-phase filler-based polymer composite at different volume concentrations.

In order to study the morphology of the APTES-TiO_2 and APTES-$BaTiO_3$ (1:3) based PVDF nanocomposite film, we have taken the surface image of it from both top (exposed with air) and bottom (attached with glass) side of the self-standing film using SEM. The SEM image of the top side reflects that the film surface is rough. It is mainly due to the evaporation of the solvent (DMF) during the annealing of the film. However, the bottom side of the film surface

is relatively smooth. The SEM image of PVDF and PVDF-TiO_2 nanocomposite based self-standing film for various volume concentrations of 5%, 10%, 20%, 30%, and 40% of TiO_2 nanoparticles of top and bottom surface are given in Figures S6 (a-f) and & S7(a-f), respectively.

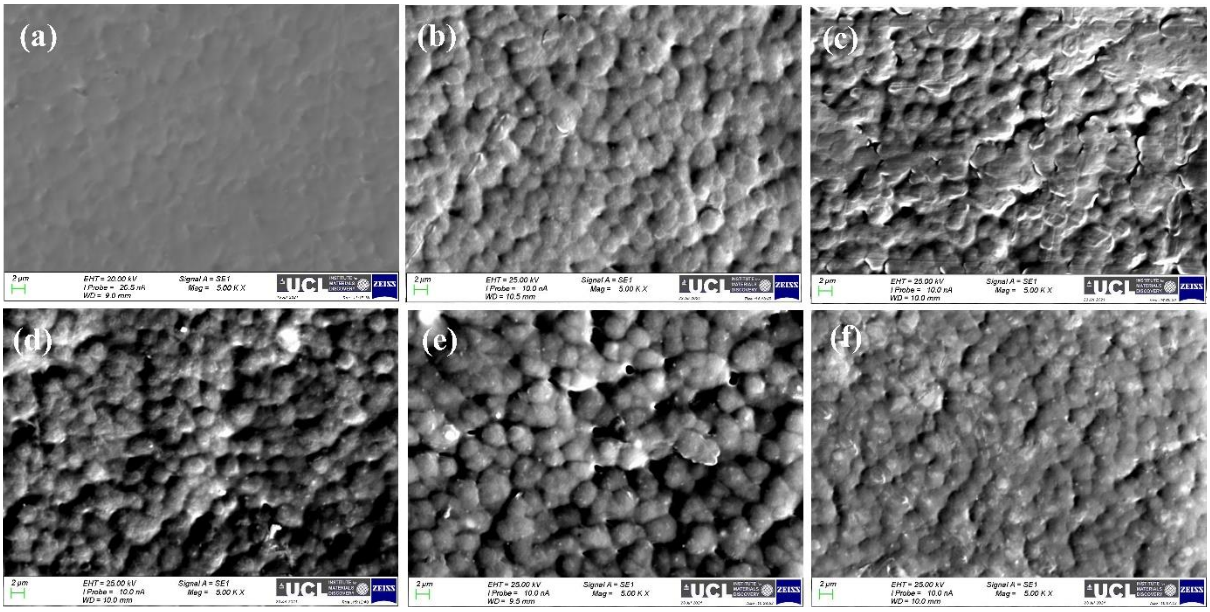

Figure S6. Surface morphology of the top surface (a) self-standing PVDF film (b-f) APTES modified TiO_2 and $BaTiO_3$ (1:3) based hybrid nanofiller-based self-standing PVDF polymer composite for 5%, 10%, 20%, 30% and 40% volume concentration of the filler materials.

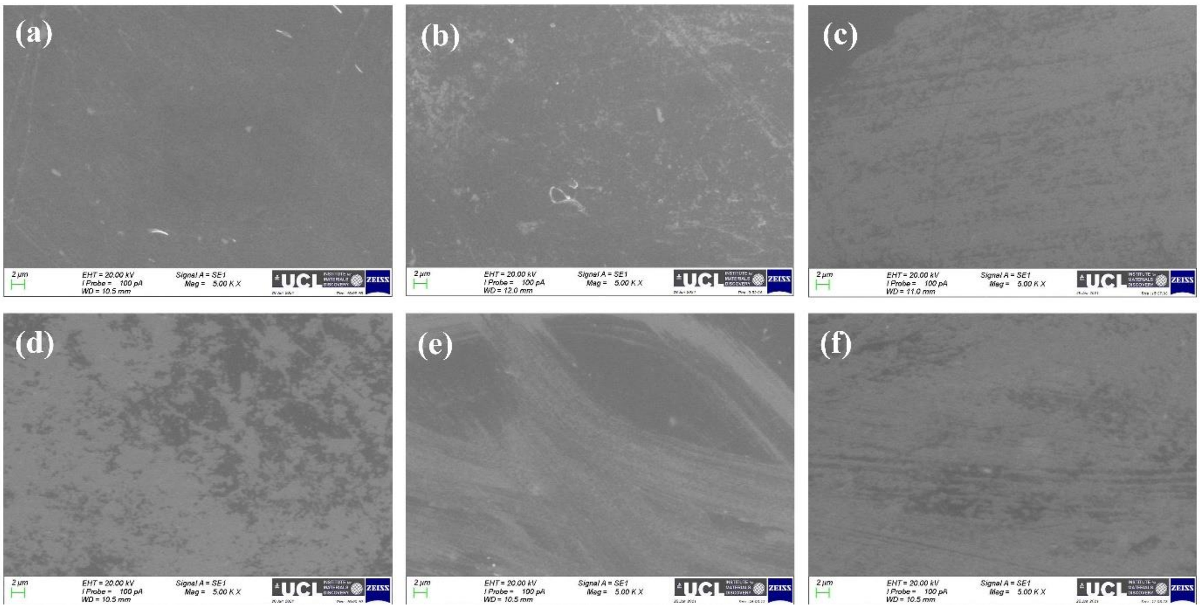

Figure S7. Surface morphology of the bottom surface (a) self-standing PVDF film (b-f) APTES modified TiO_2 and $BaTiO_3$ (1:3) based hybrid nanofiller- based self-standing PVDF polymer composite for 5%, 10%, 20%, 30% and 40% volume concentration of the filler materials, respectively.

The EDAX spectra have been taken from the APTES-modified hetero-phase filler-based composite to confirm the functionalized NPs in the film. Figure S8 shows the EDAX spectra for the PVDF film. It contains mainly carbon and fluorine, confirmed by the EDAX spectra. However, all the elements in the composite are shown in the EDAX spectra. Figure S9-S13 shows the EDAX spectra of composite materials with hetero-phase filler materials at various volume concentrations of 5%, 10%, 20%, 30% and 40%, respectively.

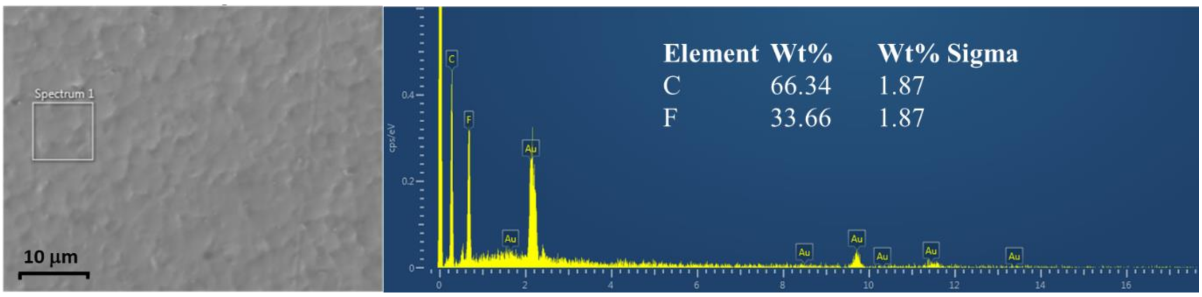

Figure S8. EDAX spectra taken from mark region in SEM image of self –standing PVDF film.

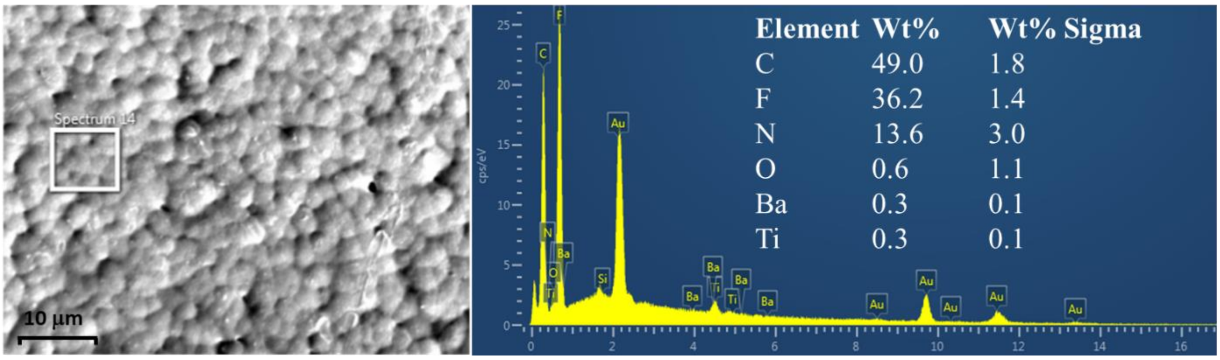

Figure S9. EDAX spectra taken from mark region in SEM image of APTES modified self–standing filler (TiO_2:$BaTiO_3$ = 1:3) based of self –standing PVDF film 5% volume concentration of filler materials.

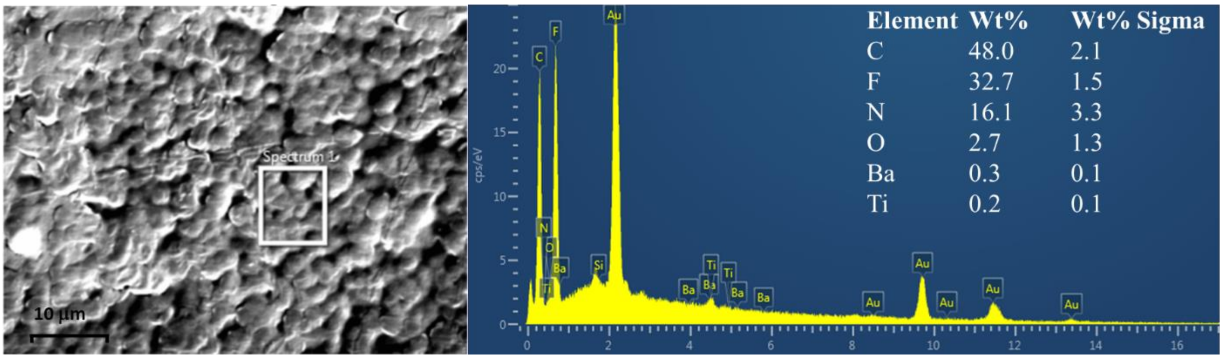

Figure S10. EDAX spectra taken from mark region in SEM image of APTES modified hetero-phase filler (TiO_2:$BaTiO_3$ = 1:3) based of self –standing PVDF film with 10% volume concentration of filler materials.

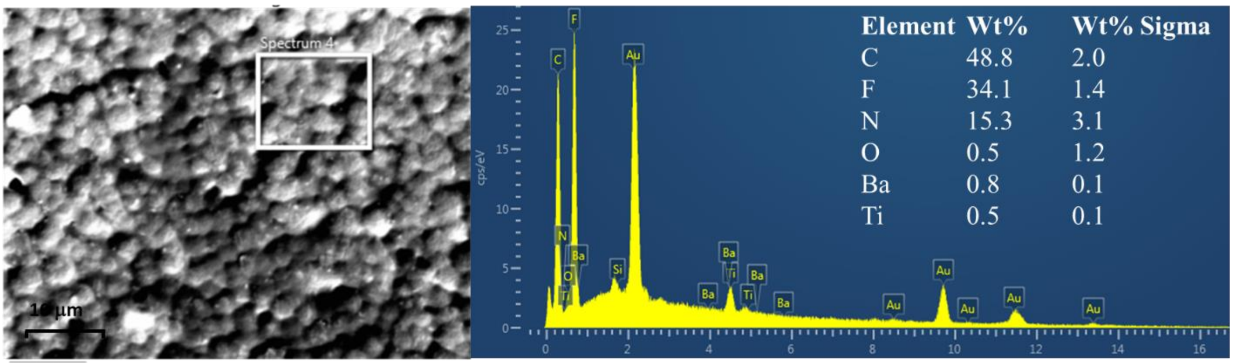

Figure S11. EDAX spectra taken from mark region in SEM image of APTES modified hetero-phase filler (TiO_2:$BaTiO_3$ = 1:3) based of self –standing PVDF film with 20% volume concentration of filler materials.

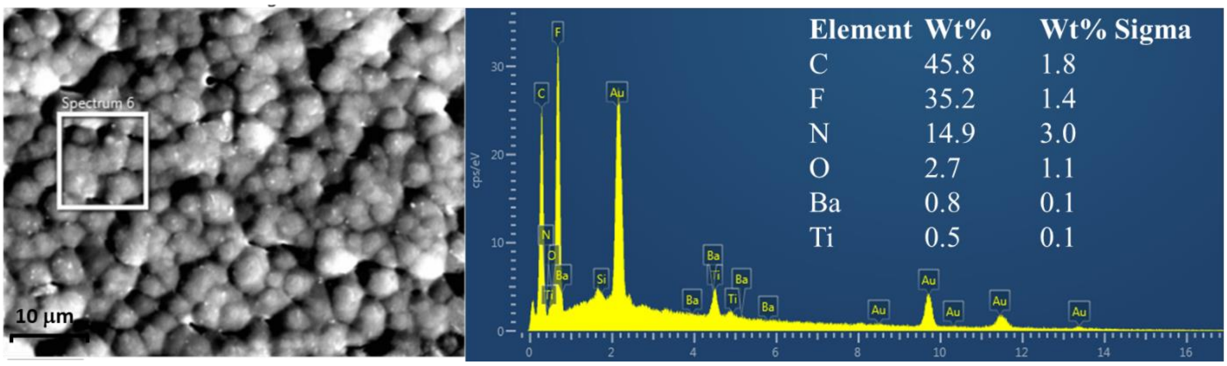

Figure S12. EDAX spectra taken from mark region in SEM image of APTES modified hetero-phase filler (TiO_2:$BaTiO_3$ = 1:3) based of self –standing PVDF film with 30% volume concentration of filler materials.

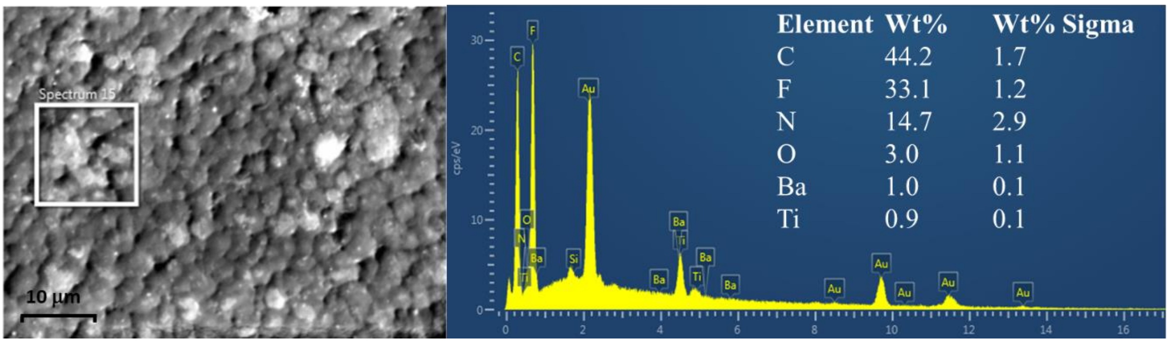

Figure S13. EDAX spectra taken from mark region in SEM image of APTES modified hetero-phase filler (TiO_2:$BaTiO_3$ = 1:3) based of self –standing PVDF film with 40% volume concentration of filler materials.

We have studied the mechanical property of PVDF and APTES-modified hetero-phase filler based PVDF composite. The stress-strain plot for PVDF and PVDF composite are shown in Fig 14a and Fig 14b, respectively. It seems after 5 vol% of 3:1 $BaTO_3$:TiO_2 NPs incorporation, the nanocomposite demonstrates a little bit higher Young's modulus (10720.189 vs 9878.869 Mpa) but lower extension (1.888 mm vs 2.218 mm).The addition of 5 vol% inorganic NPs into PVDF increased the strength a little but decreased the toughness of the polymer due to the agglomeration of NPs in the matrix.

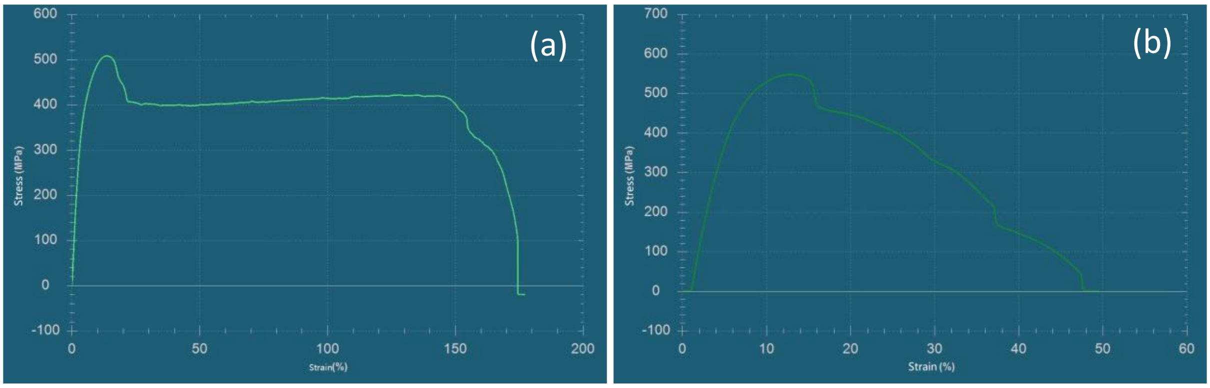

Figure S14. Mechanical test of (a) PVDF (b) APTES-modified 5 % vol. of 3:1 $BaTO_3$:TiO_2 NPs based PVDF composite.

We have compared the dielectric property of the reported PVDF nano-composite in table S1.

**Polymer matrix**	**Fillers**				**Dielectric constant at 1 kHz**	**Loss tangent at 1 kHz**	**Year**	**ref.**
	**Filler name**	**Functionalization agent**	**Diameter or length (nm)**	**content**				
Molten polypropylene grafted maleic anhydride	Barium ($BaTiO_3$) titanate and Boron Nitride (BN)	3-glycidoxypropyltrimethoxysilane.	<100 nm <100 nm	-	4.2	0.02	2022	[2]
Polyvinylidene-fluoride (PVDF	$CaCu_3Ti_4O_{12}$ (CCTO) nano-particles	Ethylenediamine (EDA)	-	8% vol	Without functionalized (14) With functionalized (19)	0.027 for both functionalized and without functionlized	2022	[3]
Polyimide (PI)	MAX (Ti_3AlC_2) nanosheets	Dopamine		7wt%	4.5	0.003	2022	[4]

Polydicyclopentadiene (PDCPD	$BaTiO_3$	Norbornene	<100 nm	35wt%	8 (only PDCPD) 19(PDCPD_35BTOH) 21(PDCPD_35BTNA)	0.001 (only PDCPD) 0.055(PDCPD_35BTOH) 0.029(PDCPD_35BTNA)	2022	[5]
Bisphenol-A epoxy resin	$BaTiO_3$	3-glycidoxypropyltrimethoxysilane	<100 nm	10Vol%	3.4 (epoxy) 5 Epoxy composite	0.004 (epoxy) 0.007 (epoxy composite)	2021	[6]
Poly(arylene ether nitrile) (PEN)	$BaTiO_3$	γ-aminopropyl triethoxysilane (KH550)	<100 nm<br><br>Average 60 nm distribution given	40wt%	10.2	0.038	2021	[7]
Carboxyl group modified Polymethyl vinyl siloxane (PMVS)	Titanium dioxide (TiO_2)	γ-(2,3-epoxypropoxy) Propytrimethoxysilane (KH560, 99%) modified	400 nm		10.2	0.04	2020	[8]
Polyvinylidene-fluoride (PVDF)	$BaTiO_3$	Dopamine modified	45	10wt%	~19	~0.04	2020	[9]
Poly(arylene ether nitrile)(PEN)	$BaTiO_3$	Dopamine and silane coupling agent KH550	177 nm	40wt%	10.7	0.11	2019	[10]
Low-density polyethylene (LDPE).	TiO_2 nanoparticles, rutile phase		30 nm	10wt%	3.1		2019	[11]
Polyvinylidene-fluoride (PVDF)	$BaTiO_3$	H_2O_2 and ethyl alcohol	137 nm	30wt%	50	0.03	2019	[12]
Polyvinylidene-fluoride (PVDF)	TiO_2−$BaTiO_3$−TiO_2 (TiO_2−BT−TiO_2@dopa) core@double-shell nanoparticles	Polydopamine (dopa)-functionalized	TiO_2 (21 nm)	10Vol%	TiO_2-PVDF (11) TiO_2@dopa/PVDF (12) TiO_2-BT@dopa	TiO_2-PVDF (0.05) TiO_2@dopa/PVDF (~0.05) TiO_2-BT@dop	2019	[13]

					/PVDF(12.2) TiO_2-BT-TiO_2@dopa/PVDF(12.6)	a/PVDF(~0.05) TiO_2-BT-TiO_2@dopa/PVDF (~0.05)		
Acrylonitrile−butadiene rubber	TiO_2	Poly(catechol/polyamine) (PCPA) and γ-(2,3-epoxypropoxy)-propytrimethoxysilane (GPTMS)	100 nm		15	0.1	2019	14
Poly(arylene ether nitrile)(PEN)	$BaTiO_3$	polyaniline-functionalized	BaTiO3 (<100 nm)	40wt%	14	0.03	2018	15
Polystyrene (PS)	$BaTiO_3$	H_2O_2 and palmitic acid (PAc)	30-150 nm	20Vol%	2.7(PS) 12(PS composite)	0.001(PS) 0.6(PS composite)	2018	16
Polyarylene ether nitrile (PEN)	$BaTiO_3$	Copper tetra-amine phthalocyanine (NH2-CuPc) and carboxyl-functionalized polyarylene ether nitrile (CPEN)	60 nm	20wt%	9.5	0.037	2017	17
Poly(vinylidene fluoride-co-hexafluoropropylene) (P(VDF-HFP)	$BaTiO_3$ NWs.	Dopamine derivative		15% Vol	23	0.018	2017	18
Polyarylene ether nitrile (PEN)	$BaTiO_3$	Carboxyl-functionalized polyarylene ether nitrile	80±10 nm	40wt%	15.2	0.022	2017	19
Polyvinylidene-fluoride (PVDF)	Strontium titanate ($SrTiO_3$)	PVP	110	40-Vol%	33.9	0.05	2017	20
Natural rubber (NR)	$BaTiO_3$	H_2O_2	100 nm		20	5	2017	21

Polyvinyl Chloride (PVC)	TiO_2	Vinyl silane	21 nm	5wt%	6	0.15	2017	[22]
Silicone rubber	$BaTiO_3$	Poly(dopamine) (PDA) and g-methacryloxypropyl trimethoxy silane (KH570)	100 nm		4.1	0.003	2016	[23]
Epoxy resin (EPR)	$BaTiO_3$	γ-aminopropyl trimethoxy silane functionalized	70 nm	20wt%	7.8(composite) 3(Epoxy)	0.002(composite) 0.004(Epoxy)	2016	[24]
Poly(vinylidene fluoride) (PVDF)	$Ba_{0.6}Sr_{0.4}TiO_3$ nanofibers (BST)	Isopropyl dioleic(dioctyl-phosphate) titanate (NDZ 101)-functionalized	Diameter 120 nm Length 10µm	7.5Vol %	21	0.05	2016	[25]
P(VDF-HFP)	$BaTiO_3$	H_2O_2	~100 nm	50%Vol	45	0.06	2015	[26]
Poly(vinylidene fluoride-co-hexafluoropropylene) [PVDF-HFP]	$BaTiO_3$	Glycidyl methacrylate (GMA) Silane	100 nm	50%Vol	42	0.03	2014	[27]
Polyvinylidene-fluoride (PVDF)	$BaTiO_3$	3-aminopropyltriethoxysilane (APS)	Diameter 100-150 nm Length 10µm	7.5Vol %	24	0.018	2014	[28]
Poly(vinylidene fluoride-co-hexafluoropropylene) P(VDF-HFP)	$CaCu_3Ti_4O_{12}$	2,3,4,5,6-pentafluorobenzyl phosphonic acid		50 Vol%	P(VDF+HFP) – 5<br><br>CCTO_ P(VDF+HFP) composite – 23<br><br>Functionalized CCTO_ P(VDF+HFP) composite - 26	CCTO_ P(VDF+HFP) composite –0.2<br><br>Functionalized CCTO_ P(VDF+HFP) composite – 0.15	2014	[29]

Polyvinylidene-fluoride (PVDF)	$BaTiO_3$	Tetrafluropthtalic acid	100 nm	50 – Vol%	40	0.02	2013	[30]
Elastomer matrix (hydrogenated nitrile-butadiene rubber	$BaTiO_3$	Poly(dopamine) (PDA)		50 phr(parts per hundred parts of rubber	15.5	0.02	2013	[31]
Polyvinylidene-fluoride (PVDF)	$BaTiO_3$	NXT-105	100	50 – Vol%	53.9	0.03	2013	[32]
Poly(amic acid)	$BaTiO_3$	H_2O_2	70 nm	50% Vol	58	0.032	2012	[33]
Polyvinylidene-fluoride (PVDF)	$BaTiO_3$	H_2O_2	85-100 nm	-	30	0.1	2011	[34]
Polyarylene ether nitrile (PEN)	$BaTiO_3$ double-layer core/shell-structured	N-(γ-aminoethyl)-γ-aminopropyltrim-ethoxy silane and hyperbranched cop-per phthalocyanine (HBCuPc)	60 nm		6.2	0.02	2011	[35]
Epoxy resin	$BaTiO_3$	GPTMS	75 nm	60%	~50		2009	[36]
PVDF	$BaTiO_3$	γ-aminopropyltriethoxy silane (KH550)	700 nm	1 wt%	~52	0.03	2006	[37]
PVDF	$BaTiO_3$ and TiO_2	APTES	<100 nm	5% Vol	76.1	0.01	2022	**This Work**

Table S1: Comparison of dielectric properties of various polymer composite materials.

References

1. Cai, X.; Lei, T.; Sun, D.; Lin, L. A critical analysis of the α, β and γ phases in poly(vinylidene fluoride) using FTIR. *RSC Adv*. **2017**, *7*, 15382.

2. Uyor, U.; Popoola, A. P. I.; Popoola, O. M.; Aigbodion, V. S., Thermal, mechanical and dielectric properties of functionalized sandwich BN-$BaTiO_3$-BN/polypropylene nanocomposites. *J. Alloys Compd.* **2022**, *894*, 162405.

3. Kaur, S.; Singh, D. P., Significantly improved dielectric and energy storage behavior of the surface functionalized $CaCu_3Ti_4O_{12}$ nanoparticles in PVDF-$CaCu_3Ti_4O_{12}$ nanocomposites. *J. Alloys Compd.* **2022**, *918*, 165500.

4. Feng, Q. K.; Dong, Q.; Zhang, D. L.; Pei, J. Y.; Dang, Z. M., Enhancement of high-temperature dielectric energy storage performances of polyimide nanocomposites utilizing surface functionalized MAX nanosheets. *Compos Sci Technol.* **2022**, *218,* 109193.

5. Chen, L.; Wang, P.; Yang, L.; Ling, J. C.; Liu, C.; Wang, Y.; Zhang, S. S.; Feng, S. J.; Wu, X. S.; Xu, P.; Ding, Y. S., Enhanced dielectric properties of high glass transition temperature PDCPD/$BaTiO_3$ composites by frontal ring-opening metathesis polymerization. *Mater. Lett.* **2022**, *310*, 131492.

6. Zafar, R.; Gupta, N., Estimation of interface properties in epoxy-based barium titanate nanocomposites. *J. Phys. Commun*. **2021**, *5*, 075003.

7. Zeng, J. J.; Tang, J. Y.; Jin, T. T.; Pu, L. Y.; Wei, X. H.; Huang, X.; Liu, J. S., Enhanced energy density of poly(arylene ether nitriles) composites filled with surface engineered $BaTiO_3$ nanoparticles. *Sens. Actuator A Phys.* **2020**, *315*, 112185.

8. Liu, X. Y.; Sun, H. B.; Liu, S. T.; Jiang, Y. J.; Yu, B.; Ning, N. Y.; Tian, M.; Zhang, L. Q., Mechanical, dielectric and actuated properties of carboxyl grafted silicone elastomer composites containing epoxy-functionalized TiO_2 filler. *Chem. Eng. J* **2020**, *393*.

9. Mayeen, A.; Kala, M. S.; Sunija, S.; Rouxel, D.; Bhowmik, R.N.; Thomas, S.; Kalarikkal, N.; Flexible dopamine-functionalized $BaTiO_3$/$BaTiZrO_3$/$BaZrO_3$-PVDF ferroelectric nanofibers for electrical energy storage. *J. Alloys Compd.* **2020**, *837*, 155492.

10. Zhan, Y. Q.; Zhang, J. M.; He, S. J.; Zhao, S. M.; Bai, Y. L.; Liu, X. B., Thermally stable and dielectric nanocomposite based on poly(arylene ether nitrile) and $BaTiO_3$ functionalized by modified mussel-inspired route. *J. Polym. Res.* **2019**, *26*, 77.

11. Wang, W. W.; Li, S. T., Improvement of Dielectric Breakdown Performance by Surface Modification in Polyethylene/TiO_2 Nanocomposites. *Materials* **2019**, *12* (20), 3346.

12. Tabhane, G. H.; Giripunje, S. M.; Kondawar, S. B., Intensifying energy density, dielectric and mechanical properties of electroactive β-PVDF/f-BTO nanocomposites. *Physica B* **2019**, *571*, 149-161.

13. Bhunia, R. P.; Siddiqui, S.; Garg, A.; Gupta, R. K., Significantly Enhanced Energy Density by Tailoring the Interface in Hierarchically Structured TiO_2-$BaTiO_3$-TiO_2 Nanofillers in PVDF-Based Thin-Film Polymer Nanocomposites. *ACS Appl. Mater. Interfaces* **2019**, *11* (15), 14329-14339.

14. Kong, X. X.; Yang, D.; Ni, Y. F.; Hao, J.; Guo, W. L.; Zhang, L. Q., Enhanced Actuation Strains of Rubber Composites by Combined Covalent and Noncovalent Modification of TiO_2 Nanoparticles. *Ind. Eng. Chem. Res.* **2019**, *58* (43), 19890-19898.

15. You, Y.; Wang, Y. J.; Tu, L.; Tong, L. F.; Wei, R. B.; Liu, X. B., Interface Modulation of Core-Shell Structured $BaTiO_3$@polyaniline for Novel Dielectric Materials from Its Nanocomposite with Polyarylene Ether Nitrile. *Polymers* **2018**, *10* (12), 1378.

16. Piana, F.; Cacciotti, I.; Slouf, M.; Nanni, F.; Pfleger, J., One-pot preparation of surface-functionalized barium titanate nanoparticles for high-K polystyrene composite films prepared via floating method. *J. Mater. Sci.* **2018**, *53* (16), 11343-11354.

17. You, Y.; Han, W. H.; Tu, L.; Wang, Y. J.; Wei, R. B.; Liu, X. B., Double-layer core/shell-structured nanoparticles in polyarylene ether nitrile-based nanocomposites as flexible dielectric materials. *Rsc Adv.* **2017**, *7* (47), 29306-29311.

18. Wang, G. Y.; Huang, X. Y.; Jiang, P. K., Bio-Inspired Fluoro-polydopamine Meets Barium Titanate Nanowires: A Perfect Combination to Enhance Energy Storage Capability of Polymer Nanocomposites. *ACS Appl. Mater. Interfaces* **2017**, *9* (8), 7547-7555.

19. Pu, Z. J.; Zheng, X. Y.; Tian, Y. H.; Hu, L. Q.; Zhong, J. C., Flexible Ultrahigh-Temperature Polymer-Based Dielectrics with High Permittivity for Film Capacitor Applications. Polymers **2017**, *9* (11) 596.

20. Wang, J.; Liu, S.; Wang, J.; Hao, H.; Zhao, L.; Zhai, J. Improving dielectric properties and energy storage performance of poly(vinylidenefluoride) nanocomposite by surface-modified $SrTiO_3$ nanoparticles. *J. Alloys Compd.* **2017**, *726*, 587- 592.

21. Gonzalez, N.; Riba, J. R.; Custal, M. D. A.; Armelin, E., Improvement of Insulation Effectiveness of Natural Rubber by Adding Hydroxyl-Functionalized Barium Titanate Nanoparticles. *IEEE Trans Dielectr Electr Insul.* **2017**, *24* (5), 2881-2889.

22. Abdel-Gawad, N. M. K.; El Dein, A. Z.; Mansour, D. E. A.; Ahmed, H. M.; Darwish, M. M. F.; Lehtonen, M., Enhancement of Dielectric and Mechanical Properties of Polyvinyl Chloride Nanocomposites Using Functionalized TiO_2 Nanoparticles. *IEEE Trans Dielectr Electr Insul.* **2017**, *24* (6), 3490-3499.

23. Yang, D.; Ruan, M. N.; Huang, S.; Wu, Y. B.; Li, S. X.; Wang, H.; Ao, X.; Liang, Y. F.; Guo, W. L.; Zhang, L. Q., Dopamine and silane functionalized barium titanate with improved electromechanical properties for silicone dielectric elastomers. *RSC Adv***.** **2016**, *6* (93), 90172-90183.

24. Phan, T. T. M.; Chu, N. C.; Luu, V. B.; Xuan, H. N.; Martin, I.; Carriere, P., The role of epoxy matrix occlusions within $BaTiO_3$ nanoparticles on the dielectric properties of functionalized BaTiO3/epoxy nanocomposites. *Compos. Part A Appl. Sci. Manuf.* **2016**, *90*, 528-535.

25. Pan, Z. B.; Yao, L. M.; Zhai, J. W.; Liu, S. H.; Yang, K.; Wang, H. T.; Liu, J. H., Fast discharge and high energy density of nanocomposite capacitors using $Ba_{0.6}Sr_{0.4}TiO_3$ nanofibers. *Ceram. Int.* **2016**, *42* (13), 14667-14674.

26. Luo, H.; Zhang, D.; Jiang, C.; Yuan, X.; Chen, C.; Zhou, K., Improved Dielectric Properties and Energy Storage Density of Poly(vinylidene fluoride-co-hexafluoropropylene) Nanocomposite with Hydantoin Epoxy Resin Coated $BaTiO_3$. *ACS Appl. Mater. Interfaces* **2015**, *7* (15), 8061-8069.

27. Xie, L. Y.; Huang, X. Y.; Yang, K.; Li, S. T.; Jiang, P. K., "Grafting to" route to PVDF-HFP-GMA/$BaTiO_3$ nanocomposites with high dielectric constant and high thermal conductivity for energy storage and thermal management applications. *J Mater. Chem. A* **2014**, *2* (15), 5244-5251.

28. Liu, S. H.; Xue, S. X.; Zhang, W. Q.; Zhai, J. W., Enhanced dielectric and energy storage density induced by surface-modified $BaTiO_3$ nanofibers in poly(vinylidene fluoride) nanocomposites. *Ceram. Int.* **2014**, *40* (10), 15633-15640.

29. Ehrhardt, C.; Fettkenhauer, C.; Glenneberg, J.; Munchgesang, W.; Leipner, H. S.; Diestelhorst, M.; Lemm, S.; Beige, H.; Ebbinghaus, S. G., A solution-based approach

to composite dielectric films of surface functionalized $CaCu_3Ti_4O_{12}$ and P(VDF-HFP). *J Mater. Chem. A* **2014**, *2* (7), 2266-2274.

30. Yu, K.; Niu, Y.; Xiang, F.; Zhou, Y. C.; Bai, Y. Y.; Wang, H., Enhanced electric breakdown strength and high energy density of barium titanate filled polymer nanocomposites. *J. Appl. Phys.* **2013**, *114* (17) 174107.

31. Yang, D.; Tian, M.; Li, D. D.; Wang, W. C.; Ge, F. X.; Zhang, L. Q., Enhanced dielectric properties and actuated strain of elastomer composites with dopamine-induced surface functionalization. *J Mater. Chem. A* **2013**, *1* (39), 12276-12284.

32. Ke Yu, H. W., Yongcun Zhou, Yuanyuan Bai, and Yujuan Niu, Enhanced dielectric properties of $BaTiO_3$/poly(vinylidene fluoride) nanocomposites for energy storage applications. *J. Appl. Phys.* **2013**, *113*, 034105.

33. Choudhury, A., Preparation, characterization and dielectric properties of polyetherimide nanocomposites containing surface-functionalized $BaTiO_3$ nanoparticles. *Polym Int.* **2012**, *61* (5), 696-702.

34. Zhou, T.; Zha, J. W.; Cui, R. Y.; Fan, B. H.; Yuan, J. K.; Dang, Z. M., Improving dielectric properties of $BaTiO_3$/ferroelectric polymer composites by employing surface hydroxylated $BaTiO_3$ nanoparticles. *ACS Appl. Mater. Interfaces* **2011**, *3* (7), 2184-2188.

35. Tang, H. L.; Ma, Z.; Zhong, J. C.; Yang, J.; Zhao, R.; Liu, X. B., Effect of surface modification on the dielectric properties of PEN nanocomposites based on double-layer core/shell-structured $BaTiO_3$ nanoparticles. *Colloids Surf. A: Physicochem. Eng. Asp.* **2011**, *384* (1-3), 311-317.

36. Iijima, M.; Sato, N.; Lenggoro, I. W.; Kamiya, H. Surface modification of $BaTiO_3$ particles by silane coupling agents in different solvents and their effect on dielectric properties of $BaTiO_3$/epoxy composites. *Colloids Surf. A: Physicochem. Eng. Asp.* **2009**, *352*, 88-93.

37. Dang, Z.M.; H.-Y. Wang, H. Y.; Xu, H. P. Influence of silane coupling agent on morphology and dielectric property in $BaTiO_3$/polyvinylidene fluoride composites. *Appl. Phys. Lett.* **2006**, *89*, 112902.
